# LncRNA *Tuna* is activated in cadmium-induced placental insufficiency and drives the NRF2-mediated oxidative stress response

**DOI:** 10.3389/fcell.2023.1151108

**Published:** 2023-06-01

**Authors:** Mark D. Simmers, Dereje D. Jima, Yoshiaki Tsuji, Michael Cowley

**Affiliations:** ^1^ Center for Human Health and the Environment, Department of Biological Sciences, North Carolina State University, Raleigh, NC, United States; ^2^ Bioinformatics Research Center, North Carolina State University, Raleigh, NC, United States

**Keywords:** lncRNA, *Tuna*, placental insufficiency, fetal growth restriction (FGR), Cadmium, Nrf2, oxidative stress

## Abstract

Cadmium (Cd) is a toxic heavy metal found throughout the environment and one of the top ten toxicants of major public health concern identified by the World Health Organization. *In utero* Cd exposure causes fetal growth restriction, malformation, and spontaneous abortion; however, the mechanisms by which Cd impacts these outcomes are poorly understood. Cd accumulates in the placenta, suggesting that these negative outcomes may be a consequence of disrupted placental function and placental insufficiency. To understand the impact of Cd on gene expression within the placenta, we developed a mouse model of Cd-induced fetal growth restriction through maternal consumption of CdCl_2_ and performed RNA-seq on control and CdCl_2_ exposed placentae. The top differentially expressed transcript was the *Tcl1 Upstream Neuron-Associated* (*Tuna*) long non-coding RNA, which was upregulated over 25-fold in CdCl_2_ exposed placentae. *Tuna* has been shown to be critical for neural stem cell differentiation. However, within the placenta, there is no evidence that *Tuna* is normally expressed or functional at any developmental stage. To determine the spatial expression of Cd-activated *Tuna* within the placenta, we used *in situ* hybridization as well as placental layer-specific RNA isolation and analysis. Both methods confirmed the absence of *Tuna* expression in control samples and determined that Cd-induced *Tuna* expression is specific to the junctional zone. Since many lncRNAs regulate gene expression, we hypothesized that *Tuna* forms part of the mechanism of Cd-induced transcriptomic changes. To test this, we over-expressed *Tuna* in cultured choriocarcinoma cells and compared gene expression profiles to those of control and CdCl_2_ exposed cells. We demonstrate significant overlap between genes activated by *Tuna* overexpression and genes activated by CdCl_2_ exposure, with enrichment in the NRF2-mediated oxidative stress response. Herein we analyze the NRF2 pathway and show that *Tuna* increases *NRF2*/NRF2 both at the transcript and protein levels. *Tuna* drives increased NRF2 target gene expression, a result that is abrogated with the use of an NRF2 inhibitor, confirming that *Tuna* activates oxidative stress response genes through this pathway. This work identifies the lncRNA *Tuna* as a potential novel player in Cd-induced placental insufficiency.

## Introduction

Declared as one of the top ten environmental toxicants of public health concern by the World Health Organization, Cd is toxic to almost all forms of life (Rahimzadeh et al., 2017). Cd occurs naturally in the Earth’s crust at low levels; however, anthropogenic and natural activity release it into the atmosphere where it can reach toxic concentrations in air, water, and soil (Rahimzadeh et al., 2017). Health effects of chronic Cd exposure include the development of cancer, kidney disease, rapid bone demineralization, reproductive dysfunction, diabetes, and osteoporosis, as well as other metabolic, cardiovascular, and behavioral disorders (Satarug et al., 2010). With a biological half-life of up to 30 years, Cd can accumulate in the kidneys and liver (Gerhardsson et al., 2012). Negative outcomes associated with *in utero* Cd exposure include fetal growth restriction (FGR), malformation, and spontaneous abortion (Kippler et al., 2010). FGR, affecting 3%–7% of pregnancies, is defined by failure to reach growth potential due to placental dysfunction resulting in low micronutrient and gas transport to the fetus (Burton and Jauniaux, 2018). During pregnancy Cd accumulates in the placenta and is inefficiently transferred to the fetus, indicating that its impacts on fetal development may be a result of placental impairment (Osman et al., 2000). While the effects of Cd on development have long been established, the mechanisms underlying Cd-induced placental dysfunction are poorly understood.

The most common cause for FGR is placental insufficiency (Salavati et al., 2019). The placenta is a crucial interface for providing gas and nutrients to the growing fetus from the maternal blood supply (Burton and Jauniaux, 2015), and placental insufficiency is described as when improper formation or damage negatively impacts this function (Gagnon, 2003). In mice, the placenta is composed of three layers: decidua, junctional zone (JZ), and labyrinth (Lab) (Watson and Cross, 2005). The decidua is composed of fetal extravillous trophoblasts that have invaded the maternal endometrium to restructure maternal spiral arteries, allowing for placental access to maternal blood supplies (Simmons et al., 2007; Silva and Serakides, 2016). The JZ is primarily composed of glycogen trophoblasts (GlyTs), which serve as energy storage, and spongiotrophoblasts (SpTs), which synthesize and secrete endocrine hormones for the placenta and fetus (Eaton et al., 2020). The Lab is composed of syncytiotrophoblasts (SynT) that line the highly vascularized basal layer enabling gas and nutrient exchange to the fetus (Simmons and Cross, 2005). Impairment to placentation and placental development are one of the main causes of stillbirths, preterm births, preeclampsia, and FGR (Gibbins et al., 2020). In this study we examine the transcriptional profiles of placentae associated with Cd-induced FGR.

Long non-coding RNAs (lncRNAs) are >200 bp long transcripts with no protein coding capabilities. They can interact with mRNA, DNA, protein, and miRNA to regulate epigenetic, transcriptional, post-transcriptional, translational, and post-translational pathways (Zhang et al., 2019). At a cellular level, lncRNAs have been shown to influence trophoblast migration, proliferation, and invasion (Muys et al., 2016; Pengjie et al., 2018; Feng et al., 2021). In addition, several lncRNAs have been associated with conditions such as pre-eclampsia and intrauterine growth restriction (Gremlich et al., 2014; Zhang et al., 2015). In humans, relationships have been found between Cd exposure, lncRNA transcript abundance in the placenta, and low birthweights (Hussey et al., 2020).

In a previous study, we established a mouse model of developmental Cd exposure which caused FGR and morphological changes to the placenta (Simmers et al., 2022). Briefly, both male and female embryos exposed to 50 ppm CdCl_2_ through maternal drinking water from 5 weeks prior to mating until collection at embryonic day 18.5 (e18.5) displayed significantly decreased weights. Placental weights expressed as a proportion of total embryo weights were significantly decreased in female embryos indicating placental insufficiency. Both male and female CdCl_2_ exposed placentae showed a significantly increased ratio of Lab to JZ signifying a major disruption to placental morphology. Herein we examine the differentially expressed genes (DEGs) between CdCl_2_ exposed and control placentae through RNA-seq. We identify the lncRNA *Tuna* as the most differentially expressed gene and use an *in vitro* overexpression model to demonstrate that it drives the nuclear factor erythroid 2-related factor 2 (NRF2) oxidative stress response pathway, suggesting that *Tuna* may be a key part of the cellular response to Cd. Our findings have implications for understanding the mechanisms through which Cd causes placental dysfunction and FGR.

## Methods

### Animal model and tissue collection

Mice were maintained on a 14-h/10-h light/dark cycle at 30%–70% humidity, 22°C ± 4°C. Mating, exposure, and collection of whole placentae and layer-specific tissue were performed as previously described (Simmers et al., 2022). In short, 5-week-old female C57BL/6J mice were exposed for 5 weeks to 0 or 50 ppm CdCl_2_ through drinking water (Sigma-Aldrich, 202908). In this model, 5 weeks of exposure to 50 ppm CdCl_2_ results in blood Cd levels in dams of 5.23 ± 0.99 μg/L (mean ± standard error) (Hudson et al., 2019), similar to the blood Cd levels of ∼6 μg/L found in people living in a contaminated community in Japan (Sasaki et al., 2019). At 10 weeks of age, mice were mated with unexposed CAST/EiJ males. The use of divergent parental strains facilitated the analysis of imprinted genes in our previous study (Simmers et al., 2022). CdCl_2_ exposure continued throughout mating and gestation. Placentae and fetuses were isolated at e18.5; collected tissues were weighed, snap-frozen immediately upon dissection and stored at −80°C or prepared for histological analysis as described below.

### Nucleic acid isolation

Eight female placental samples representing at least four litters from independent dams were selected from each treatment group for nucleic acid isolation. Tissues were homogenized in lysis buffer and RNA was isolated via AllPrep DNA/RNA/miRNA kit (Qiagen). RNA from layer-specific tissues was isolated via AllPrep DNA/RNA Micro kit (Qiagen). RNA from Jeg-3 cells was isolated via NucleoSpin RNA kit (Macherey-Nagel). Nucleic acids were quantified on a Nanodrop 2000 and RNA integrity was confirmed by electrophoresis.

### qRT-PCR

500 ng of total RNA from whole placenta, 100 ng of layer-specific total RNA, or 500 ng of total RNA from Jeg-3 cells was used to synthesize first strand cDNA according to the manufacturer’s protocol (M-MLV RT enzyme, Promega) (Simmers et al., 2022). qRT-PCR was performed in triplicate on 96-well plates with a QuantStudio 3 Real-Time PCR system (Applied Biosystems) using SsoAdvanced Universal SYBR Green Supermix (Bio-Rad). The cycling conditions were as follows: 95°C for 30 s; 40 cycles of 95°C for 15 s, 60°C for 30 s; dissociation curve of 60.0°C–95.0°C. The primer sequences are provided in Supplementary Table S1. Mouse *Polr2a* or human *GAPDH* were used as reference genes and were not significantly differentially expressed between treatment groups (data not shown). Gene expression was quantified using the ΔΔCt method (Livak and Schmittgen, 2001). *Tuna/TUNA* expression was quantified using the ΔCt method to determine its expression as a percentage of the housekeeper (*Polr2a/GAPDH*).

### Cell culture

Jeg-3 cells were purchased from the American Type Culture Collection (Manassas, VA) and maintained in minimum essential media (Corning Life Sciences) supplemented with 10% FBS (Genesee Scientific) and 100 μg/mL Streptomycin, 100 U/mL Penicillin (HyClone), and humidified in 5% CO_2_ at 37°C.

### CdCl_2_, PbAc, and NaAsO_2_ exposure

Cells were seeded in 6-well plates for 24 h at 4 × 10^5^ cells/well until reaching ∼80% confluency. Cells were starved with serum free media for 24 h at which point serum free media was aspirated, cells were washed in PBS, and exposed to 0, 0.5, 1.0, 2.5, 5.0, and 10.0 µM concentrations of CdCl_2_, PbAc, or NaAsO_2_ in serum free media. Each exposure was done in triplicate, cells were collected after 24 h of exposure, and each experiment was repeated 3 times. Viability was determined using CytoTox-Glo (Promega) according to the manufacturer’s instructions.

### Transfection

Cells were seeded in 6-well plates for 24 h at 4 × 10^5^ cells/well until reaching ∼80% confluency. Expression of *GFP* (control) or *Tuna* was achieved by transfecting cells with 3.5 μg of pcopGFP or pcopGFP-mTuna using Lipofectamine 3000 according to the manufacturer’s protocol (ThermoFisher). PcopGFP-mTuna was created by inserting the mouse *Tuna* sequence into the multiple cloning site (MCS) of pCDH-CMV-MCS-EF1α-GreenPuro Cloning and Expression Lentivector (System Biosciences). To remove the *Tuna* sequence and generate the pcopGFP control vector, the pcopGFP-mTuna construct was cut at the EcoRI and BamHI restriction sites, blunted with T4 DNA polymerase and ligated with T4 DNA ligase (New England Biolabs). Each condition was done in triplicate, transfected cells were collected 24, 48, or 72 h post transfection, and all experiments were repeated 3 times.

### *In situ* hybridization

*Fresh Frozen*–In a cryostat, fresh frozen e18.5 placentae samples were partially embedded in OCT compound (Tissue-Tek), sectioned (20 µm thickness) and fixed in 10% formalin.

*Formalin-Fixed Paraffin-Embedded (FFPE)*—Placentae were collected at e18.5 and fixed in 4% formaldehyde for 24 h. Within 24 h of fixation, placentae were delivered in 70% ethanol to the NC State Histology Laboratory for dehydration and paraffin embedding. Samples were then sectioned to 20 µm and pretreated according to the Formalin-Fixed Paraffin-Embedded (FFPE) Sample Preparation and Pretreatment protocol by Advanced Cell Diagnostics.

All samples were then dehydrated, dried, treated, and probed using the RNAscope 2.5 HD assay according to the manufacturer’s protocol (ACD) using Mm-Tunar-O1 (REF#579711) or Mm-Polr2a (REF#312471) probes. The protocol was modified to limit protease exposure to 15 min, and a 30 s eosin counterstain step was added directly after the hematoxylin stain and ammonia water rinse.

### Viability and proliferation assays

Cell viability and proliferation assays for transfected cells were conducted using the trypan blue exclusion test (Strober, 2015). Cells were grown and exposed or transfected as described above. All conditions were done in triplicate, viability was checked after 24, 48, and 72 h post transfection, and each experiment was repeated 3 times. At each time point the ratio of living cells to total cells was used to calculate percent viability and total cell count was used for proliferation.

### H_2_O_2_ detection

Levels of H_2_O_2_ were determined via ROS-Glo H_2_O_2_ Kit (Promega). Cells were seeded in 96-well plates at 1 × 10^4^ cells/well and cultured overnight until reaching ∼80% confluency. Expression of *GFP* (control) or *Tuna* was achieved as described above. Cells were exposed to 50 µM menadione for 2 h to induce oxidative stress as a positive control. Each condition was done in triplicate. Transfected, control, and menadione treated cells were analyzed at 24 h, and all experiments were repeated 3 times.

### RNA-seq

RNA was isolated from e18.5 female mice or Jeg-3 cells as described above. Integrity, purity, and concentration were determined via the Agilent 2100 Bioanalyzer with an RNA 6000 Nano Chip (Agilent Technologies, United States). All samples had an RNA integrity number (RIN) > 9.0. Samples were submitted to the NC State University Genomic Sciences Laboratory for Illumina RNA library construction and sequencing. cDNA libraries for Illumina sequencing were constructed via the NEBNext Ultra Directional RNA Library Prep Kit (NEB) and NEBNext Multiplex Oligos for Illumina (NEB) according to the manufacturer’s protocol. Samples were sequenced on the Illumina NovaSeq S4, utilizing a 150 × 2 bp paired end S4 sequencing reagent kit (Illumina).

Data analysis was performed in consultation with the Bioinformatics Core at NC State University’s Center for Human Health and the Environment. An average of ∼61 million paired-end raw RNA-seq reads were generated for each replicate. The quality of sequenced data was assessed using the *fastqc* application, and 12 poor-quality bases were trimmed from the 5’-end. The remaining good-quality reads were aligned to the mouse or human reference genome (mm10 version 87 or GRCh38 respectively) databases using the STAR aligner (Dobin et al., 2013). To validate single nucleotide polymorphisms (SNPs) in the hybrid mouse model, the following WASP options were passed to the STAR aligner (*--varVCFfile C57BL_6NJ.mgp.v5.snps.dbSNP142.vcf--waspOutputMode SAMtag*). Per-gene counts of uniquely mapped reads for each replicate were calculated using the *htseq-count* script from the HTSeq (Anders et al., 2015) Python package. The count matrix was imported to R statistical computing environment for further analysis. Initially, genes that had no count in greater than half of the replicate samples were discarded. The remaining count data were normalized for sequencing depth and distortion, and dispersion was estimated using DESeq2 (Love et al., 2014) Bioconductor package. We fitted a linear model using the treatment levels, and differentially expressed genes (DEGs) were identified after applying multiple testing corrections using the Benjamini–Hochberg procedure (Benjamini and Hochberg, 1995)⁠⁠. The final list of significant genes was generated using an adjusted *p*-value <0.05. RNA-seq data are deposited in the Gene Expression Omnibus (accession number GSE233282).

### Enrichment analysis

For gene set enrichment analysis, the common DEGs between the 0 vs. 5 µM CdCl_2_ exposed Jeg-3 cells and pcopGFP vs. pcopGFP-mTuna transfected cells detected through RNA-seq were analyzed via hypergeometric calculation as described previously (Baptissart et al., 2022).

#### Ingenuity Pathway Analysis (IPA)

Ensembl IDs of significant DEGs were input for core analysis using Ingenuity Pathway Analysis (IPA) software (Qiagen). For all analyses, the ‘Upstream regulators’ were filtered to only consider the molecule type ‘Genes, RNAs and proteins’.

### Western blot

Cells were exposed or transfected as previously described and washed with PBS and lysed in ice-cold RIPA buffer (ThermoFisher) with protease inhibitor cocktail (Sigma-Aldrich). Protein concentration was determined with the BCA Protein Assay Kit (Pierce) and samples were boiled with 4X Laemmli buffer for 5 min. Equal amounts of protein were resolved in 4%–15% gradient SDS-PAGE gels (BioRad) and transferred to polyvinylidene difluoride membranes (Merck Millipore). Membranes were cut according to target molecular weight and probed with anti-NRF2 (Abcam, #137550), TXNRD1 (Abcam, #124954), or GAPDH (Abcam, #245355) overnight on a shaker at 4°C. Antibody dilutions are presented in Supplementary Table S2. Membranes were washed 3X in 5% non-fat milk for 15 min each before incubating for 1 h on a shaker at room temperature in secondary antibody (Abcam, #7090). Finally, membranes were washed 3X in TBST for 15 min each, incubated in enhanced chemiluminescence reagents (ThermoFisher), and imaged with the AI600 (GE). Band density analysis was performed using Photoshop (Adobe).

### Statistical analysis

All statistical analyses unless otherwise specified were performed on values determined through median absolute deviation (MAD) analysis using a one-way analysis of variance (ANOVA) or Student’s t-test, two-tailed. Data are presented as the mean ± standard error of the mean. *p < 0.05, **p < 0.01, ***p < 0.001, ****p < 0.0001. The numbers of animals, litters and samples used in each experiment are presented in Supplementary Table S2.

## Results

### Tcl1 Upstream Neuron-Associated (Tuna) lncRNA is the top differentially expressed transcript between CdCl_2_ exposed and control placentae

**FIGURE 1:**
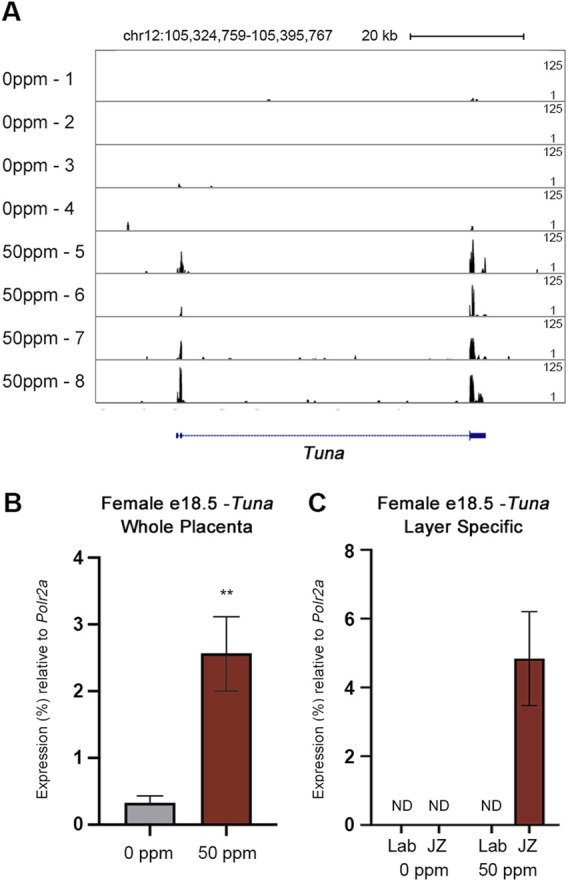
Visualization and quantification of *Tuna* transcript abundance through RNA-seq and qRT-PCR. **(A)** RNA-seq reads mapped to *Tuna* in the mm10 genome build visualized through UCSC Genome Browser. Sample names are listed on the left (four 0 ppm and four 50 ppm CdCl_2_ exposed placentae, each from unique litters). Peaks are based on a proportion of 125 maximum reads. **(B)** *Tuna* transcript abundance in e18.5 female whole placenta determined through qRT-PCR. **(C)** *Tuna* transcript abundance in e18.5 female placental Lab and JZ. Data are presented as a percentage of *Polr2a*. Data are presented as means ± SE (*n* = 8 per group). Whole placenta qRT-PCR results were analyzed by Student’s *t*-test, two-tailed test comparing 0 ppm and 50 ppm exposures. **p < 0.01. ND = not detected.

To begin to understand how Cd could impact placental function thereby modulating fetal growth, we performed RNA-seq on female control and 50 ppm CdCl_2_ exposed placentae collected from our mouse model of Cd-induced FGR (Simmers et al., 2022). Females were selected because CdCl_2_ exposure induces FGR in females without impacting placental weight; in contrast, CdCl_2_ exposed male placentae show significantly reduced weight at e16.5 although this difference does not persist at e18.5. RNA-seq revealed a total of 783 significant DEGs (adjusted *p*-value <0.05), consisting of 433 downregulated (55.3%) and 350 upregulated (44.7%) genes. Of those, *Tcl1 Upstream Neuron-Associated* (*Tuna*) lncRNA was the top differentially expressed transcript with a ∼25-fold increase in expression in CdCl_2_ exposed placentae compared to controls (Figure 1A; Supplementary Table S3). Despite being conserved in all vertebrates with a sequenced genome, *Tuna* has not been widely studied. It has been described as a crucial factor for neural stem cell differentiation, a tumor suppressor involved in breast cancer progression, an oncogene in glioma cells, and a differentiation and implantation factor in the endometrium (Lin et al., 2014; Li et al., 2017; Wang et al., 2020). However, a role for *Tuna* in the placenta or in the cellular response to an environmental stressor has not been described.

To validate the finding from RNA-seq, the abundance of placental *Tuna* transcripts was analyzed through qRT-PCR. Using this approach, CdCl_2_ exposure was associated with a 7-fold increase in placental *Tuna* transcripts compared to controls (Figure 1B). Together, our results from RNA-seq and qRT-PCR confirm *Tuna* to be poorly expressed in control placentae; however, in response to CdCl_2_ exposure, *Tuna* transcript abundance is significantly increased.

### Tuna expression is specific to the junctional zone in CdCl_2_ exposed placenta

Given the different functions performed by the layers of the placenta, we aimed to determine where in the e18.5 placenta *Tuna* is expressed in response to CdCl_2_ exposure. Using layer-specific micropunches of control and 50 ppm CdCl_2_ exposed placentae obtained and validated in our previous study, we analyzed the spatial expression of *Tuna* through qRT-PCR (Simmers et al., 2022). *Tuna* was only detectable within the JZ of the 50 ppm CdCl_2_ samples; both the JZ and Lab fractions of control placentae as well as the Lab fraction of 50 ppm CdCl_2_ exposed placentae demonstrated undetectable levels of *Tuna* transcripts (Figure 1C).

**FIGURE 2:**
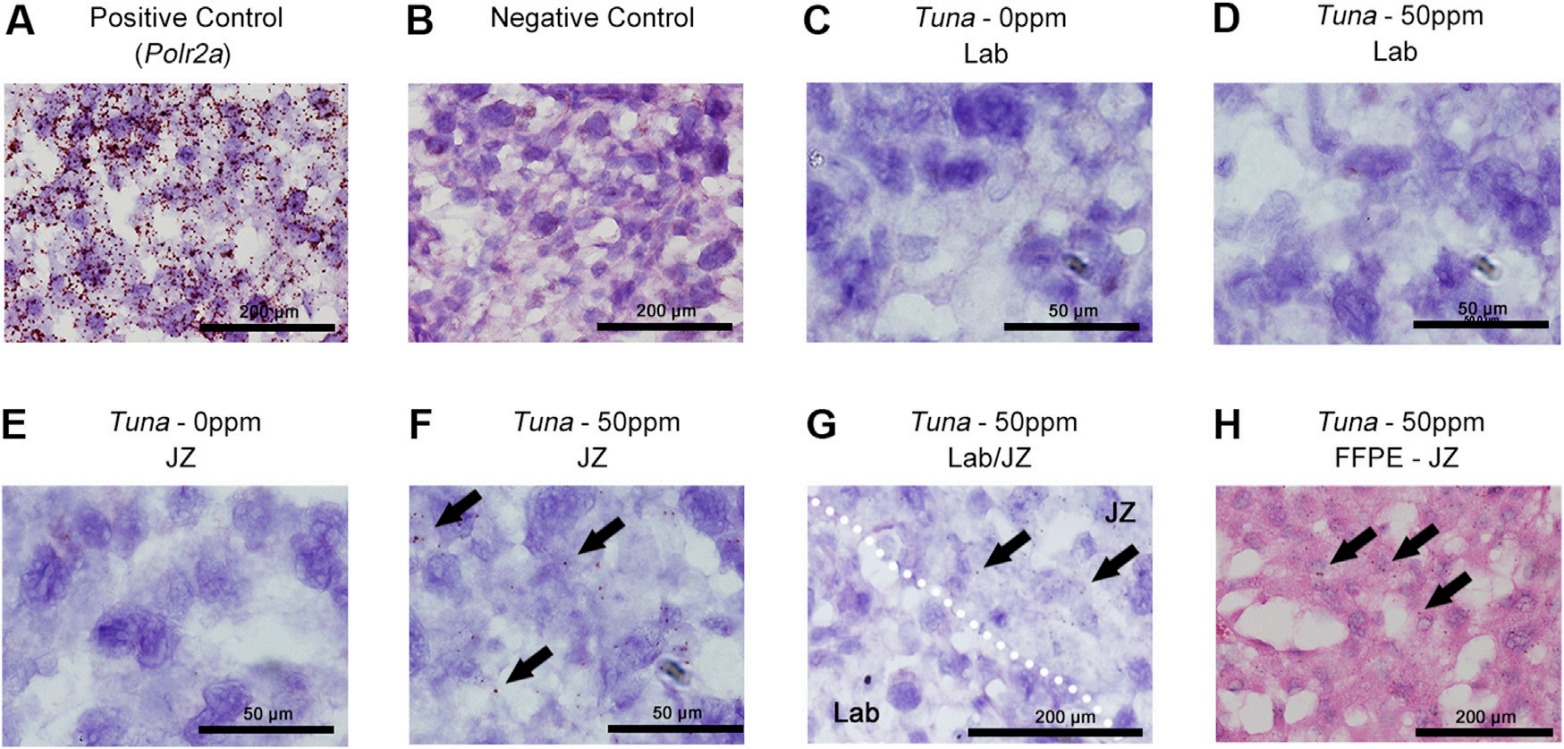
Distribution of *Tuna* transcripts in female e18.5 mouse placental sections. **(A)** Detection of positive control *Polr2a* in H&E counter-stained fresh frozen e18.5 placenta; brown signal represents at least a single *Polr2a* mRNA molecule. **(B)** Negative control. **(C)** *Tuna* in 0 ppm Lab. **(D)** *Tuna* in 50 ppm Lab. **(E)** *Tuna* in 0 ppm JZ. **(F)** *Tuna* in 50 ppm JZ. **(G)** *Tuna* at the intersection of Lab and JZ in 50 ppm CdCl_2_ sections. **(H)** *Tuna* in 50 ppm JZ formalin fixed paraffin embedded sections. Arrows indicate positive *Tuna* signal. *In situ* hybridization was performed on *n* = 4 per group; representative images are shown.

To further confirm this layer specificity and identify the cell type responsible for the increase in *Tuna* transcripts, we performed *in situ* hybridization on e18.5 placental sections. Positive (*Polr2a*) and negative controls demonstrated the expected results, validating the efficacy of the protocol (Figures 2A, B). In fresh frozen placental sections, *Tuna* transcripts were undetectable in the Lab of both control and 50 ppm CdCl_2_ exposed samples as well as control JZ samples (Figures 2C–E). In support of our qRT-PCR data, *Tuna* transcripts were detectable exclusively in the JZ of the 50 ppm CdCl_2_ exposed samples (Figure 2F). Analysis of the Lab/JZ intersection showed the dispersed signal of *Tuna* transcripts present with the JZ (Figure 2G, top right) and absence of signal in the Lab (bottom left). To obtain better cell morphology and inform on the spatial distribution of *Tuna* transcripts within the JZ, we performed *in situ* hybridization on formalin fixed paraffin embedded tissue. Consistent with our prior results, no *Tuna* signal was found in 50 ppm CdCl_2_ exposed Lab sections (Supplementary Figure S1) but was present within the JZ of 50 ppm CdCl_2_ exposed placentae including SpTs (Figure 2H).

### CdCl_2_ exposure increases Tuna expression in Jeg-3 human choriocarcinoma cells

**FIGURE 3:**
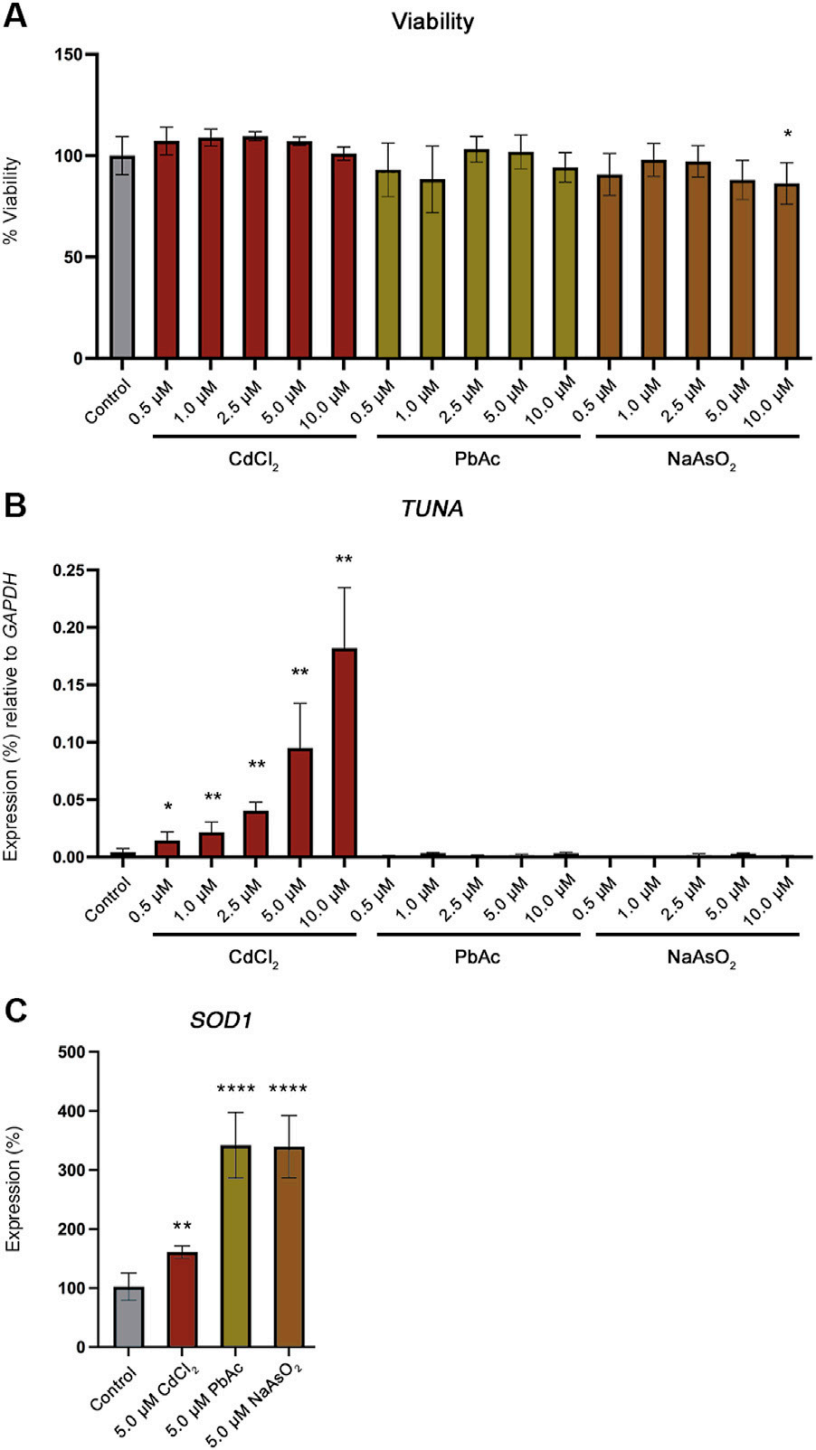
Viability and *TUNA* expression in Jeg-3 cells after 24 h of toxic metal exposure. **(A)** Viability of Jeg-3 cells exposed to 0, 0.5, 1.0, 2.5, 5.0, and 10.0 µM concentrations of CdCl_2_, PbAc, and NaAsO_2_ for 24 h, detected by CytoTox-Glo. **(B)** *TUNA* transcript abundance in Jeg-3 cells in response to 0, 0.5, 1.0, 2.5, 5.0, and 10.0 µM concentrations of CdCl_2_, PbAc, and NaAsO_2_ analyzed by qRT-PCR. **(C)** *SOD1* transcript abundance in unexposed control cells and in 5 μM CdCl_2_, 5 μM PbAc, 5 μM NaAsO_2_ exposed Jeg-3 cells at 24 h. Data are presented as mean of mean ± SE from three technical replicates within each of three independent experiments. Analyzed by one-way ANOVA with Tukey’s post-hoc test on values determined through MAD analysis. *p < 0.05, **p < 0.01, ****p < 0.0001.

We developed an *in vitro* cell model using the human choriocarcinoma cell line Jeg-3 to determine if *TUNA* is activated by Cd in a cell autonomous manner. Jeg-3 cells were exposed to 0, 0.5, 1.0, 2.5, 5.0, and 10.0 µM concentrations of CdCl_2_ for 24 h. No significant changes to cell viability were observed (Figure 3A). *TUNA* transcript abundance significantly increased in a CdCl_2_ dose dependent manner across all exposure concentrations (Figure 3B). Next, we explored whether this response was specific to CdCl_2_ or could be induced by other toxic metals. To test this, Jeg-3 cells were exposed to 0, 0.5, 1.0, 2.5, 5.0, and 10.0 µM concentrations of lead acetate (PbAc) or sodium arsenite (NaAsO_2_) for 24 h. No significant changes to viability were detected with the exception of 10.0 µM NaAsO_2_ (Figure 3A). As a control for the uptake of CdCl_2_, PbAc and NaAsO_2_, we compared the transcript abundance of the oxidative stress marker Cu/Zn Superoxide Dismutase 1 (*SOD1*). *SOD1* transcript abundance is a known marker of oxidative stress as it encodes an enzyme responsible for the conversion of superoxide radicals into molecular oxygen and hydrogen peroxide (Banks and Andersen, 2019). CdCl_2_, PbAc and NaAsO_2_ exposures all resulted in significant increases in *SOD1* expression (Figure 3C).

Neither PbAc or NaAsO_2_ significantly increased *TUNA* transcript abundance at any concentration confirming that the increase in *TUNA* expression is specific to CdCl_2_ exposure (Figure 3B).

#### Tuna overexpression causes reduced proliferation of human Jeg-3 cells

**FIGURE 4:**
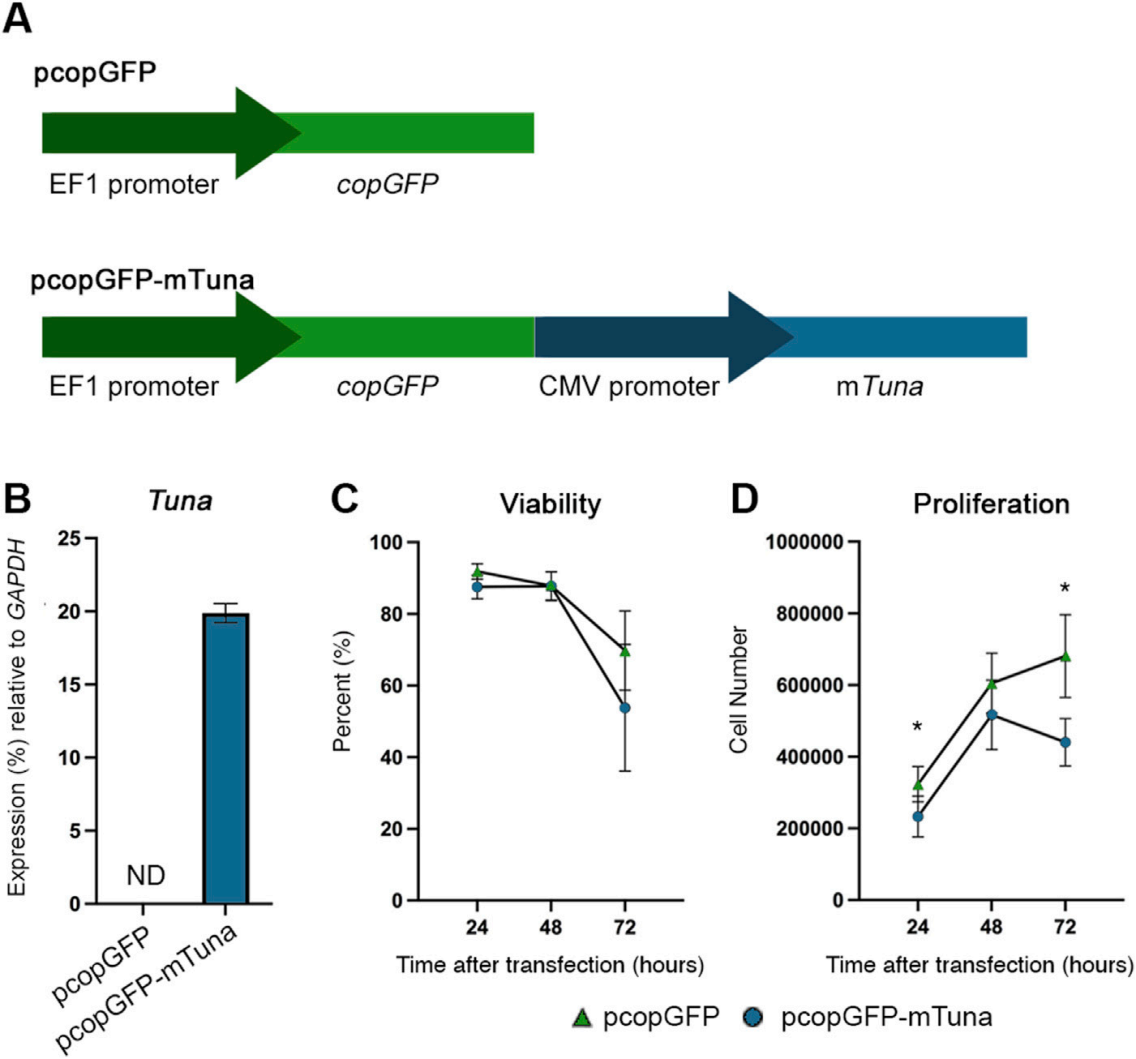
*Tuna* expression construct, viability, and proliferation assays of control vs. *Tuna* overexpressing Jeg-3 cells at 24, 48, and 72 h after transfection. **(A)** Structure of empty control vector (pcopGFP) and *Tuna* expression vector (pcopGFP-mTuna). **(B)** *Tuna* transcript abundance in Jeg-3 cells 24 h after transfection determined by qRT-PCR. **(C)** Viability of Jeg-3 cells at 24, 48, and 72 h post transfection, represented as a percent of total pcopGFP cells at 0 h. **(D)** Proliferation of Jeg-3 cells at 24, 48, and 72 h post transfection, data points represent total cell number at each time point. Experiments were performed three times, each with three technical replicates. Analyzed by Student’s *t*-test, two-tailed, comparing pcopGFP and pcopGFP-mTuna across the experimental replicates determined through MAD analysis. *p < 0.05.

To begin to study the functions of Cd-induced *Tuna* in the placenta, we created an expression construct (pcopGFP-mTuna) using the spliced mouse *Tuna* sequence detected within the placentae of our FGR model. An empty vector (pcopGFP) served as a control (Figure 4A). Constructs were transfected into Jeg-3 cells and successful transfection was determined by visualizing fluorescence from the GFP protein. *GFP* transcript abundance was measured by qRT-PCR to ensure no significant differences were detected between pcopGFP and pcopGFP-mTuna transfections within experiments (data not shown). *Tuna* expression was confirmed in cells transfected with the pcopGFP-mTuna construct (Figure 4B).

To test if *Tuna* plays a role in the response of cells to environmental exposures capable of inducing oxidative stress, we compared both viability and proliferation between pcopGFP and pcopGFP-mTuna transfected cells at 24, 48, and 72 h after transfection. There were no significant differences in viability at any time point between the two groups of cells (Figure 4C). However, proliferation was significantly decreased at 24 and 72 h in the pcopGFP-mTuna transfected cells compared to controls (Figure 4D). This suggests that Cd-induced increases in *Tuna* transcript abundance may contribute to the anti-proliferative effects associated with Cd toxicity (Erboga and Kanter, 2016).

#### Tuna overexpression activates the NRF2-mediated oxidative stress response pathway

**FIGURE 5:**
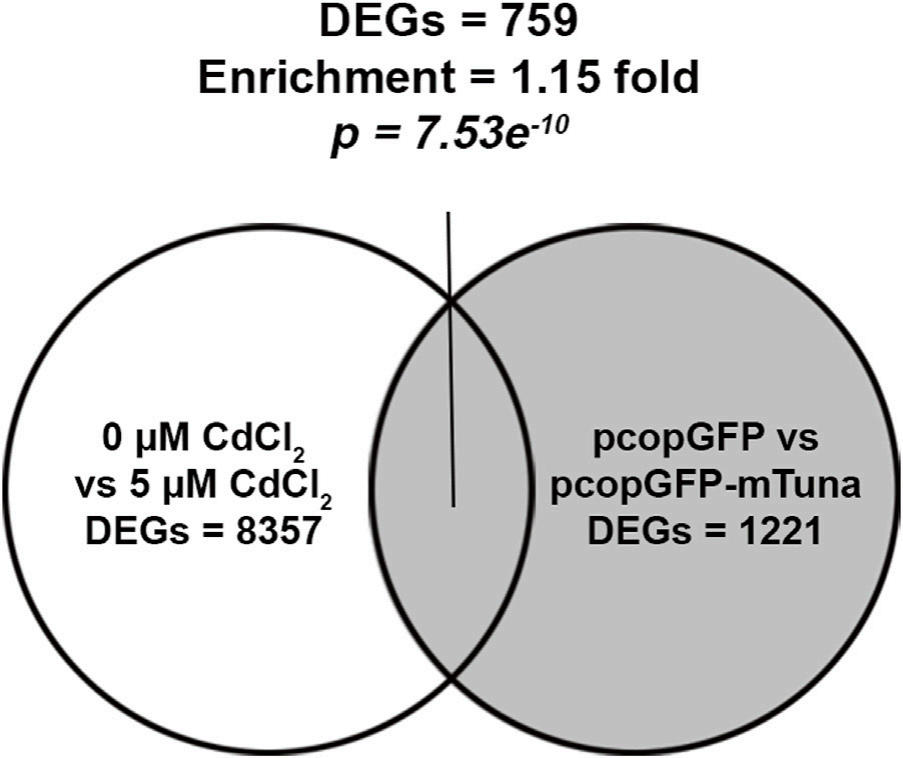
RNA-seq analysis on *Tuna* overexpressing and CdCl_2_ exposed Jeg-3 cells at 24 h. Venn diagram represents the number of CdCl_2_ DEGs and their intersection with *Tuna* overexpressing DEGs in Jeg-3 cells at 24 h exposure or post transfection. RNA-seq was performed on 4 replicates per group.

To understand the role *Tuna* might play in the cellular response to CdCl_2_, we aimed to compare RNA-seq results from CdCl_2_ exposure and *Tuna* overexpression experiments for enrichment of DEGs. RNA isolated from pcopGFP and pcopGFP-mTuna transfected Jeg-3 cells was submitted for RNA-seq alongside RNA from control (0 μM) and 5 μM CdCl_2_ exposed cells (Supplementary Tables S4, S5). RNA-seq of pcopGFP versus pcopGFP-mTuna transfected cells revealed a total of 1221 DEGs, of which 455 were downregulated (37.3%) and 766 upregulated (62.7%). RNA-seq of 0 versus 5 μM CdCl_2_ exposed cells revealed a total of 8357 DEGs, 4124 of which were downregulated (49.4%) and 4233 upregulated (50.6%). A total of 759 DEGs were shared between the two datasets, representing a statistically significant enrichment (enrichment x1.15; *p*-value = 7.53e^−10^; hypergeometric test) (Figure 5; Supplementary Table S6). This finding provides support for *Tuna* playing a role in the response to Cd exposure in the placenta. We analyzed the shared DEGs from the two datasets using Ingenuity Pathway Analysis (IPA) to identify common pathways. Oxidative phosphorylation, mitochondrial dysfunction, and NRF2-mediated oxidative stress response were the three most significantly enriched pathways (Supplementary Table S7). The NRF2-mediated oxidative stress response pathway showed the highest activation z-score indicating the pathway with the most likely activation of biological function.

Nuclear factor erythroid (NF-E2)-related factor 2 (NRF2) is a transcription factor responsible for coordinating and regulating the xenobiotic and oxidative stress response. NRF2 binds antioxidant response elements (AREs) to promote the expression of over 200 genes (Rojo de la Vega et al., 2018; He et al., 2020). In addition to its role in cellular defense, NRF2 has been associated with metabolism, inflammation, autophagy, proteostasis, mitochondrial physiology, and immune system functions (Tonelli et al., 2018; Song et al., 2021).

**FIGURE 6:**
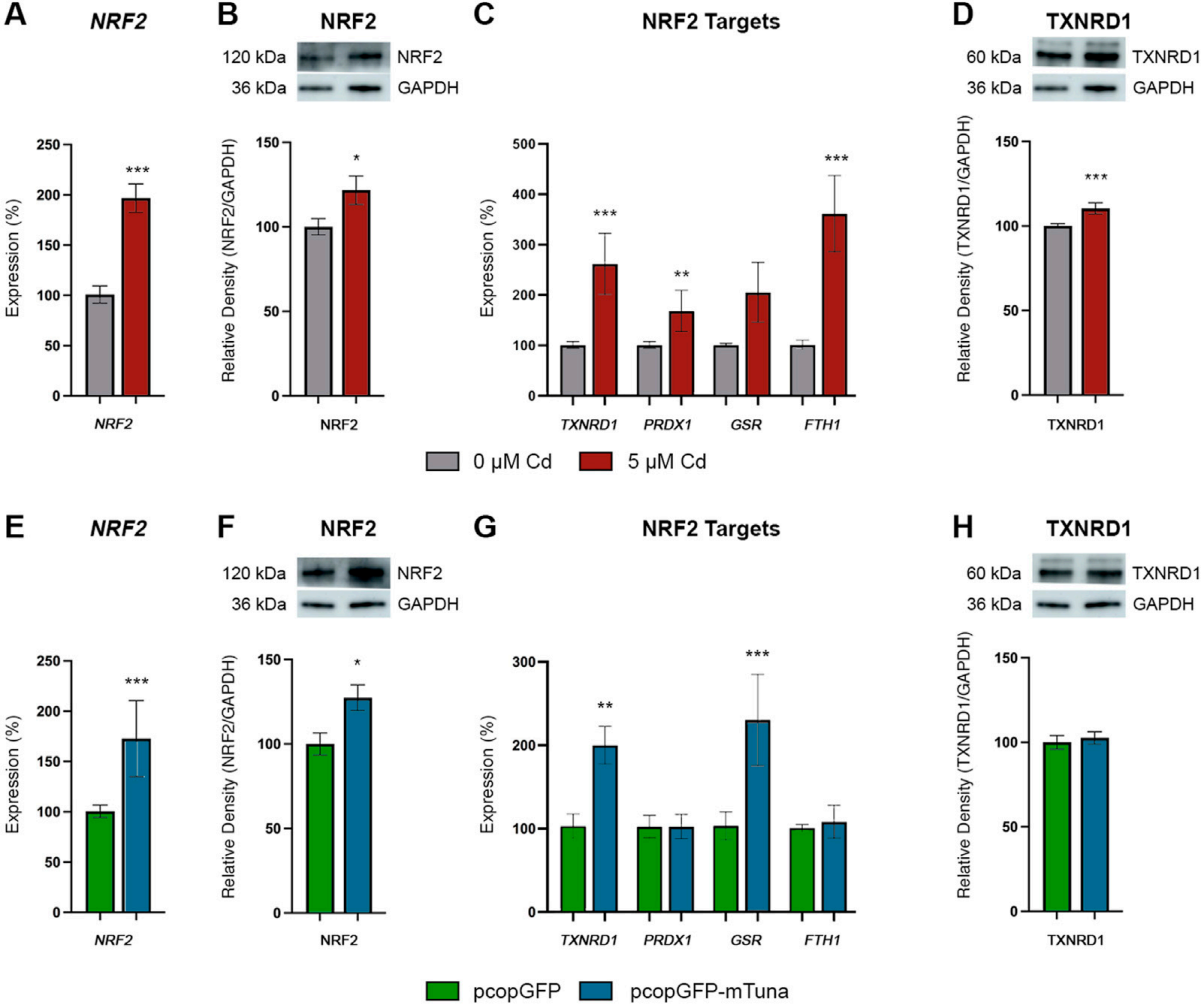
NRF2 Oxidative Stress Response Pathway transcript and protein abundance in Jeg-3 cells in response to CdCl_2_ exposure and *Tuna* overexpression. **(A)** *NRF2* transcript abundance in Jeg-3 cells in response to 0 and 5 μM CdCl_2_ at 24 h. **(B)** NRF2 protein abundance in response to 0 and 5 µM CdCl_2_ at 24 h. **(C)** *TXNRD1, PRDX1, GSR,* and *FTH1* transcript abundance in response to 0 and 5 µM CdCl_2_ at 24 h. **(D)** TXNRD1 protein abundance in response to 0 and 5 µM CdCl_2_ at 24 h. **(E)** *NRF2* transcript abundance in Jeg-3 cells in response to pcopGFP and pcopGFP-mTuna at 24 h. **(F)** NRF2 protein abundance in response to pcopGFP and pcopGFP-mTuna at 24 h. **(G)** *TXNRD1, PRDX1, GSR,* and *FTH1* transcript abundance in response to pcopGFP and pcopGFP-mTuna at 24 h. **(H)** TXNRD1 protein abundance in response to pcopGFP and pcopGFP-mTuna at 24 h. Analyzed by Student’s *t*-test, two-tailed, on values determined through MAD analysis from three independent experiments, each with three technical replicates. *p < 0.05, ***p < 0.001. Representative westerns blots are shown in panels **(B)**, **(D)**, **(F)**, and **(H)**. Membranes were cut and probed independently for NRF2, TXNRD1, and GAPDH therefore GAPDH images are duplicated between **(B)** and **(D)**, and between **(F)** and **(H)**.

To further investigate the potential role of *Tuna* in the NRF2-mediated oxidative stress response pathway, we analyzed *NRF2*/NRF2 transcript and protein levels, the transcript abundance of NRF2 targets (*TXNRD1*, *PRDX1*, *GSR*, and *FTH1*), and TXNRD1 protein abundance in Jeg-3 cells exposed to CdCl_2_ and cells overexpressing *Tuna*. We first analyzed the pathway in response to CdCl_2_. 5 μM CdCl_2_ induced significant increases of *NRF2* transcript and protein abundance compared to controls (Figures 6A, B). Similarly, the NRF2 targets *TXNRD1*, *PRDX1*, and *FTH1* were significantly increased, while *GSR* showed no significant changes (Figure 6C). Lastly, TXNRD1 protein levels were significantly increased in response to CdCl_2_ (Figure 6D). Next, we analyzed the same pathway in the context of *Tuna* overexpression. Similar to CdCl_2_ exposure, *Tuna* overexpression caused a significant increase in both NRF2 transcript and protein abundance when compared to pcopGFP controls (Figures 6E, F). In addition, *TXNRD1* and *GSR* showed increased transcript abundance while *PRDX1* and *FTH1* showed no significant changes (Figure 6G). The increased abundance of *TXNRD1* transcripts was not reflected by an increase in TXNRD1 protein abundance (Figure 6H). These results suggest that in addition to driving *NRF2* expression, *Tuna* at least partially activates the NRF2-mediated oxidative stress response pathway.

#### Tuna directly activates the NRF2 oxidative stress response pathway

**FIGURE 7:**
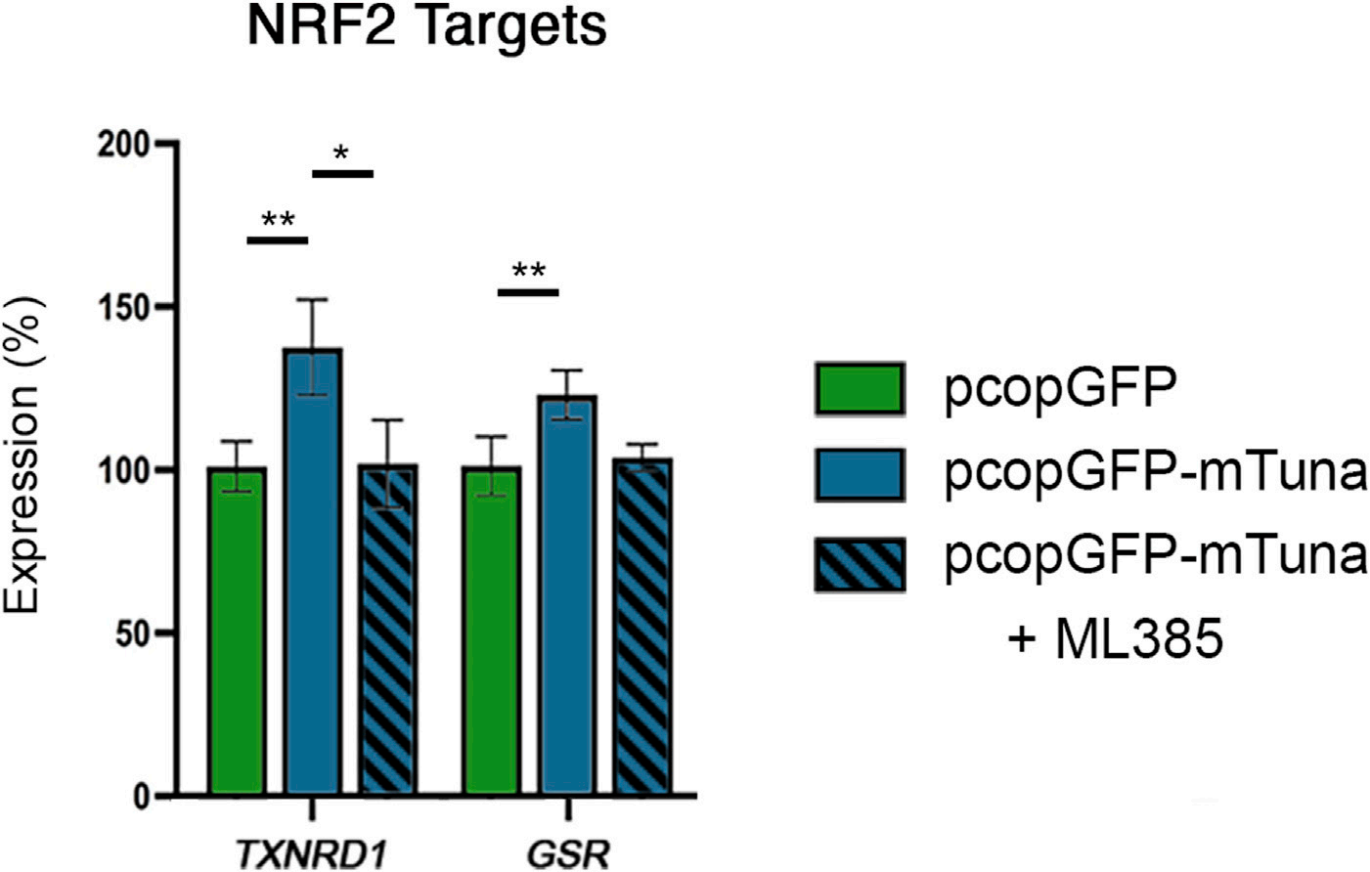
NRF2 target gene expression in response to GFP control, *Tuna* overexpression, and *Tuna* overexpression + ML385 in Jeg-3 cells 24 h post transfection. Data are presented as mean of means ± SE from three technical replicates within each of three independent experiments. Analyzed by one-way ANOVA with Tukey’s post-hoc test on values determined through MAD analysis. *p < 0.05, **p < 0.01.

The capability of lncRNAs to interact with DNA, RNA, and proteins allows them to regulate gene transcription and signaling pathways through a variety of mechanisms (Statello et al., 2021). While it is possible that *Tuna-*induced activation of selected NRF2 target genes occurs through direct binding of *Tuna* transcripts to the promoters of these genes, our observation that *Tuna* upregulates *NRF2*/NRF2 transcript and protein levels suggests that *Tuna* might drive the expression of these targets through the NRF2 pathway. To test this hypothesis, we compared NRF2 target transcript abundance between cells transfected with pcopGFP, pcopGFP-mTuna, and pcopGFP-mTuna in the presence of the NRF2 inhibitor ML385. As before, the expression of *TXNRD1* and *GSR* was significantly increased in cells overexpressing *Tuna* compared to *GFP* overexpressing controls, but this did not occur in the presence of ML385 (Figure 7). These data show that *Tuna* is acting through NRF2 to drive the NRF2-mediated oxidative stress response.

#### Tuna overexpression does not increase reactive oxygen species

**FIGURE 8:**
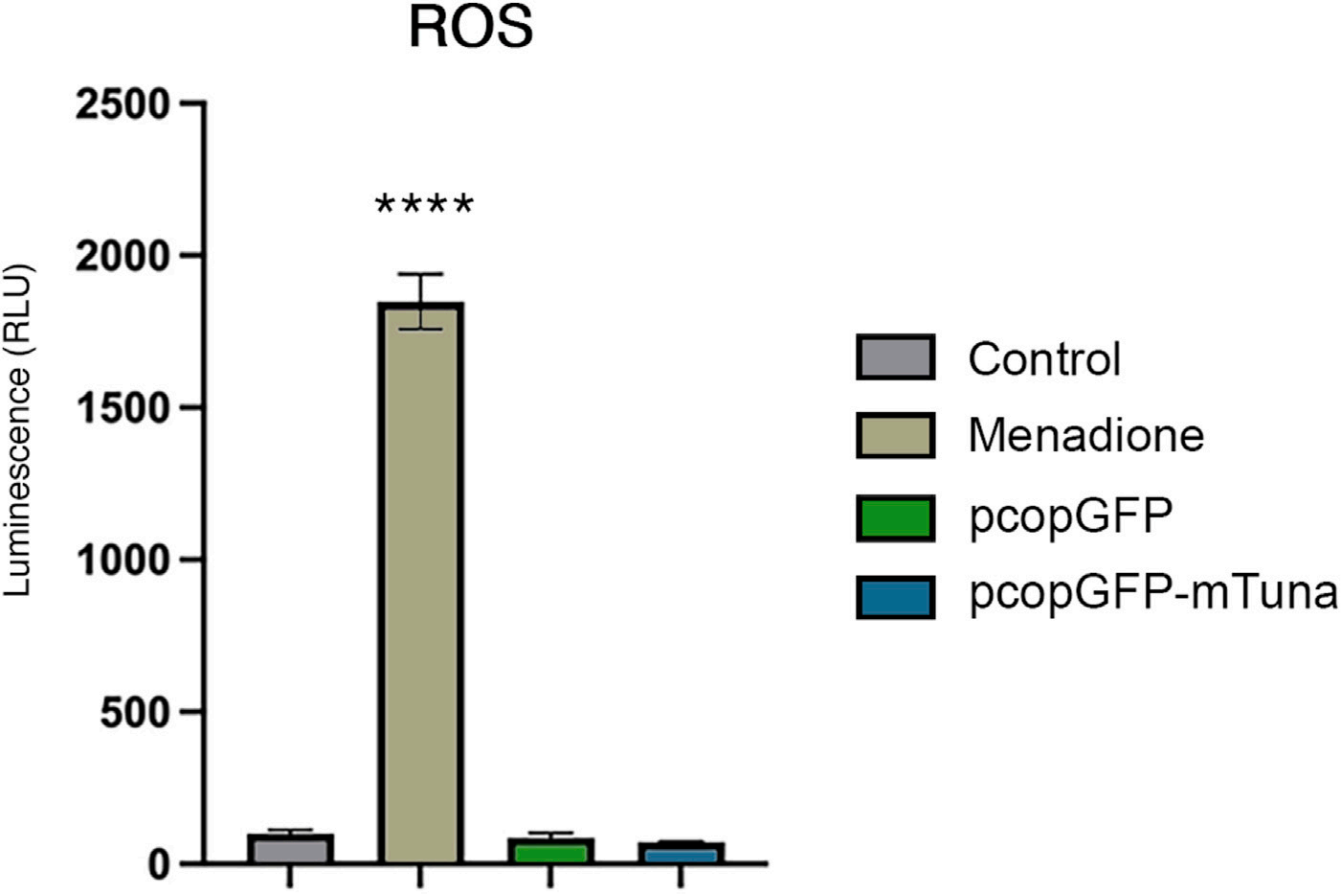
ROS levels in vehicle (control) and menadione exposed Jeg-3 cells compared to GFP control and *Tuna* overexpressing cells at 24 h post transfection. Data are presented as mean of means ± SE from three technical replicates within each of three independent experiments. Analyzed by one-way ANOVA with Tukey’s post-hoc test on values determined through MAD analysis. ****p < 0.0001.

To determine if *Tuna* drives the generation of reactive oxygen species (ROS) and thereby activates the oxidative stress response, we analyzed the H_2_O_2_ levels present in Jeg-3 cells overexpressing *Tuna* at 24 h post transfection. As a positive control for ROS, cells were treated with menadione. As expected, cells exposed to menadione displayed a significant increase in ROS compared to all other conditions (Figure 8). Neither GFP overexpressing controls nor *Tuna* overexpressing cells produced elevated levels of ROS. In addition, there was no significant difference between the ROS of GFP overexpressing controls and *Tuna* overexpressing cells, indicating that the activation of the NRF2-mediated oxidative stress response pathway in *Tuna* over-expressing cells cannot be explained by increased ROS. These results suggest that *Tuna* overexpression is driving the NRF2-mediated oxidative stress response independent of ROS.

## Discussion

FGR is a condition in which the fetus fails to reach its growth potential (Burton and Jauniaux, 2018). Affecting up to 7% of pregnancies, FGR is not only the leading cause of stillbirths and prenatal mortality but is one of the most characterized risk factors for cardiovascular disease in adult life, the leading cause of premature mortality globally (Barker, 1995; Audette and Kingdom, 2018; Crispi et al., 2018; Francula-Zaninovic and Nola, 2018; Woods et al., 2018). Placental insufficiency is one of the most common causes of FGR, and both placental dysfunction as well as FGR have been correlated with Cd exposure (Taylor et al., 2016; Burton and Jauniaux, 2018; Geng and Wang, 2019). While the mechanisms behind Cd-induced FGR and placental insufficiency are not fully understood, prior research has revealed altered expression of placental gene networks present in those with metal exposure-associated FGR (Deyssenroth et al., 2017). We hypothesized that aberrant transcript expression in the placenta caused by Cd exposure drives improper placental development leading to placental insufficiency and negative birth outcomes.

We previously established a mouse model of Cd-induced FGR wherein 50 ppm CdCl_2_ administered through drinking water before and throughout pregnancy caused significant FGR and placental insufficiency at embryonic day 18.5 (e18.5) (Simmers et al., 2022). In the current study, whole placenta RNA was isolated from control and 50 ppm exposed samples for transcriptomic analysis. Through RNA-seq we identified 783 DEGs between control and 50 ppm CdCl_2_ exposed samples. The top DEG, *Tuna*, was upregulated ∼25 fold in the placentae of offspring exposed to CdCl_2_ versus controls. qRT-PCR confirmed the increase in *Tuna* transcript abundance; however, the increase was ∼7 fold. The discrepancy between fold increases is likely due to differences in the sensitivity of the two techniques.

*Tuna*, first described in 2011 and also called ‘megamind’, is a ∼3200 bp lncRNA with high evolutionary conservation across vertebrates (Lin et al., 2014; Bouckenheimer et al., 2018). While it has been detected within the endometrium of the uterine wall potentially indicating a critical role in decidualization, no studies have identified it within the placenta at any stage of development (Wang et al., 2020). In mice, *Tuna* has been described as a crucial factor for both neural stem cell differentiation and maintenance of pluripotency through the formation of an RNA/protein complex that targets promoters of *Nanog*, *Sox2*, and *Fgf4* to induce their expression (Lin et al., 2014). *TUNA* was found to be increased in human breast cancer tissues, and *in vitro* experiments on human breast cancer cell lines suggest that *TUNA* promotes oncogenic activity through the expression of *SOX2* and promotion of epithelial-to-mesenchymal transition (Li et al., 2017). More recently, it has been revealed that *Tuna* transcripts previously thought to have no protein coding capability may encode microproteins (proteins smaller than 100 amino acids), responsible for reduced differentiation potential in mouse embryonic stem cells (Senís et al., 2021). The capability of *Tuna* to form multiple complexes and possible microproteins dependent upon developmental stage and tissue make it a potential regulatory molecule through which environmental exposures could modulate transcription. Differentially expressed lncRNAs have been associated with low birth weights and in addition Cd exposure has been shown to disrupt the expression of lncRNAs critical to fetal growth (Hussey et al., 2020).

Through the isolation of RNA from specific layers of the placenta, we determined that *Tuna* was expressed exclusively in the JZ in response to CdCl_2_ exposure, an area composed of SpTs, GlyTs and a layer of trophoblast giant cells directly bordering the decidua (Simmons et al., 2007). Identifying the specific cell type in fresh frozen sections was not possible due to the loss of clear cell morphology. However, in FFPE samples, which better preserve morphological features with the exception of GlyTs that were digested in the fixation process, we observed both cytoplasmic and nuclear signals throughout all cell types present within the JZ. This indicated that SpTs were the most likely cell type responsible for the highest *Tuna* expression. Outside of the fact that the JZ acts as an endocrine compartment maintaining progesterone secretion, little is known about the full purpose and function of SpTs within the JZ (Tanaka et al., 1997; Coan et al., 2006).

To further study the effect of CdCl_2_ on *Tuna* expression we switched to an *in vitro* model using the human choriocarcinoma cell line Jeg-3. Induction of *TUNA* expression by CdCl_2_ exposure was cell autonomous and was specific to CdCl_2_ rather than being a general response to heavy metal exposure, as no detectable changes in *TUNA* transcript were detected in response to PbAc and NaAsO_2_.

**FIGURE 9:**
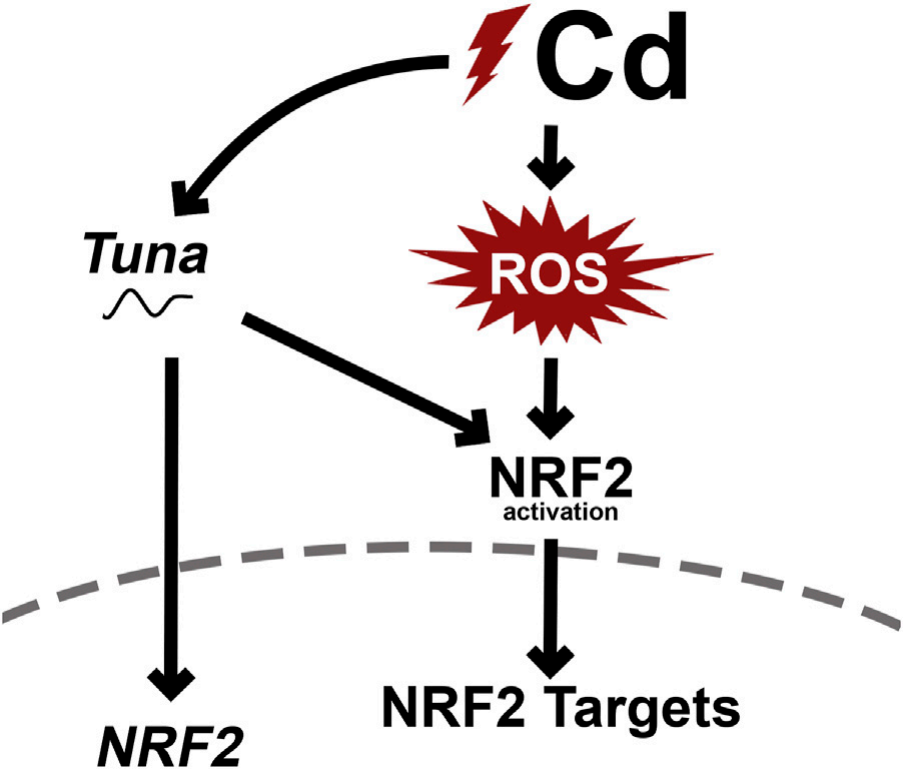
Proposed mechanism of Cd-induced *Tuna* expression and downstream molecular changes.

To further understand the role of *Tuna* in CdCl_2_ exposed Jeg-3 cells, we created a *Tuna* expression construct. While we used the mouse *Tuna* sequence in these studies, the sequence shows high evolutionary conservation with the human ortholog. Overexpression of *Tuna* activated the NRF2-mediated oxidative stress response pathway shown through increased *NRF2*/NRF2 transcript and protein abundance, as well as increased transcript abundance of several of its targets including *TXNRD1* and *GSR* as illustrated in the proposed model (Figure 9). Not all NRF2 targets analyzed displayed differential expression in response to *Tuna* overexpression. It is possible that *Tuna* is only partially activating the NRF2-mediated stress response pathway and that other cofactors only present in oxidative stress conditions lead to the increased expression of other gene targets. Alternatively, only a subset of targets may appear to be activated due to the time point analyzed. This might also account for the differences in TXNRD1 protein detection between CdCl_2_ exposure and *Tuna* overexpression.

Further, use of the NRF2 protein inhibitor ML385 showed that *Tuna* drives NRF2 target gene expression by activating the NRF2 pathway rather than independently driving the expression of NRF2 target genes. NRF2 is a critical transcription factor in the protective response to oxidative and electrophilic stress by controlling the regulation of detoxifying enzymes and antioxidant proteins (Baird and Yamamoto, 2020). NRF2 is negatively regulated by Kelch-like ECH-associated protein 1 (KEAP1), an adapter protein for ubiquitin ligase Cullin3 (CUL3)-containing E3 ubiquitin ligase complex, which retains NRF2 in the cytoplasm and enables constant ubiquitination and degradation by the ubiquitin proteasome system (UPS), maintaining low basal levels of NRF2 (Zhang et al., 2005; Villeneuve et al., 2010). Under conditions of oxidative stress NRF2 dissociates from the CUL3-KEAP1-E3 complex, translocating to the nucleus where it forms heterodimers with small musculoaponeurotic fibrosarcomas (sMAFs), binding the antioxidant response element (ARE), leading to expression of NRF2 target genes (Kaspar et al., 2000; Nam and Keum, 2019). NRF2 targets include over 200 transcripts that range from xenobiotic response, oxidative stress response, and Phase II metabolizing enzymes (Gao et al., 2021).

Our results show that both *NRF2* transcript and protein levels increase in response to *Tuna* overexpression. However, it is unknown whether the activation of the NRF2 pathway is a result of *Tuna* driving *NRF2* expression to levels outcompeting NRF2 sequestration or if *Tuna* modulates the CUL3-KEAP1-E3 complex. Further experiments are required to understand the mechanisms through which *Tuna* controls NRF2 protein levels.

Studies in rat cells have shown that CdCl_2_ activates Nrf2, protecting against Cd-induced apoptosis (Chen and Shaikh, 2009). However, our viability and proliferation assays of *Tuna* overexpression did not show any changes in viability and actually showed decreased proliferation. This may indicate that there is a protective function of *Tuna* that is acting outside of NRF2. LncRNAs have been shown to act as scaffolding for protein complexes capable of targeting enhancer and promoter regions of the genome. Utilizing an RNA/chromatin interaction assay such as chromatin isolation by RNA purification (ChIRP) would help to identify potential genomic locations to which *Tuna* binds and may reveal potential novel roles of *Tuna* in controlling the cellular response to Cd.

*Tuna* was recently shown to affect decidualization and differentiation of endometrial stromal cells (Wang et al., 2020). Patients with recurrent implantation failure (RIF), described as three failed *in vitro* fertilization attempts, showed significantly increased *Tuna* transcript levels in the endometrium compared to controls, potentially indicating a role in implantation and decidualization success (Bashiri et al., 2018). Decidualization involves the invasion of extravillous trophoblasts into the endometrium allowing for the restructuring of spiral arteries. *Tuna* may therefore play distinct roles in the endometrium and the JZ of the placenta.

These data show evidence of a novel role for *Tuna* within the placenta and present the first study to analyze the function of *Tuna* within a placental cell model. Further studies of the molecular mechanisms underlying Cd-induced *Tuna* activation and its relevance to placental insufficiency could identify new pathways underlying FGR.

## Data Availability

The datasets presented in this study can be found in online repositories. The names of the repository/repositories and accession number(s) can be found in the article/Supplementary Material.
