# Supplementary figures and images for: LncRNA Tuna is activated in cadmium-induced placental insufficiency and drives the NRF2-mediated oxidative stress response
Source: Front Cell Dev Biol. 2023 Jun 1;11:1151108. doi: 10.3389/fcell.2023.1151108 (PMC10267411; doi:10.3389/fcell.2023.1151108)

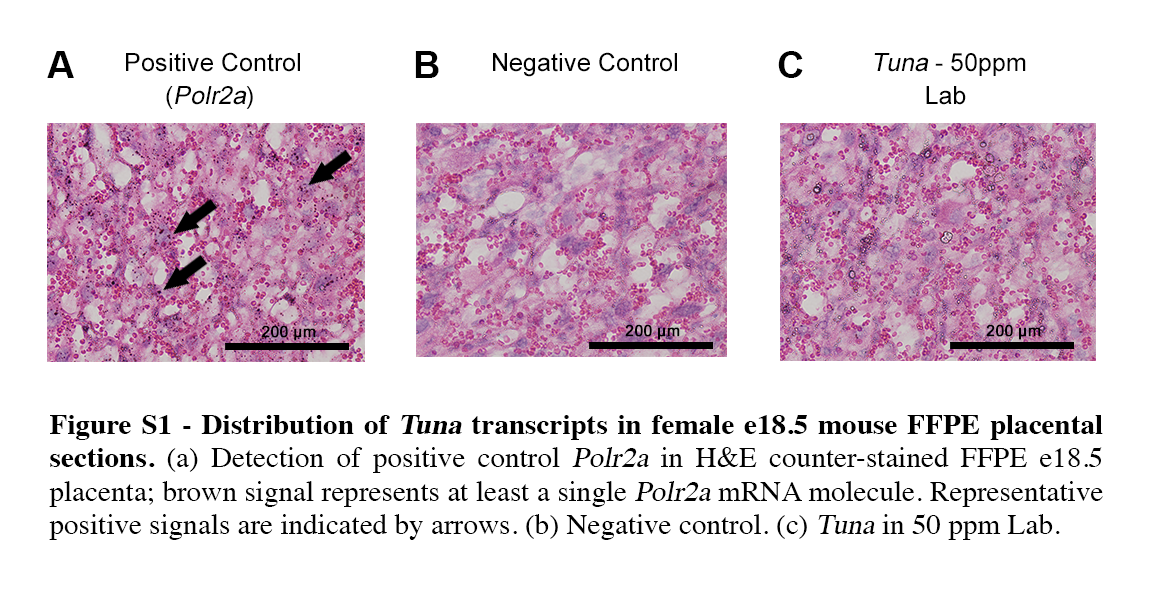

Supplement: Supplementary file 3 [file Image1.tif]
